# Expansion of highly stable *bla*_OXA-10_ β-lactamase family within diverse host range among nosocomial isolates of Gram-negative bacilli within a tertiary referral hospital of Northeast India

**DOI:** 10.1186/s13104-017-2467-2

**Published:** 2017-04-04

**Authors:** Anand Prakash Maurya, Debadatta Dhar, Mridul Kumar Basumatary, Deepjyoti Paul, Birson Ingti, Debarati Choudhury, Anupam Das Talukdar, Atanu Chakravarty, Shweta Mishra, Amitabha Bhattacharjee

**Affiliations:** 1 Department of Microbiology, Assam University, Silchar, 788011 India; 2 Department of Microbiology, Silchar Medical College and Hospital, Silchar, 788014 India; 3 Department of Life Science and Bioinformatics, Assam University, Silchar, 788011 India; 4 Department of Microbiology, Institute of Medical Sciences, Banaras Hindu University, Varanasi, 221005 India

**Keywords:** OXA-10 family, IncY, Integron, Gram-negative rods, Expanded-spectrum cephalosporins

## Abstract

**Background:**

The current study reports dissemination of highly stable *bla*_OXA-10_ family of beta lactamases among diverse group of nosocomial isolates of Gram-negative bacilli within a tertiary referral hospital of the northern part of India.

**Methods:**

In the current study, a total number of 590 Gram negative isolates were selected for a period of 1 year (i.e. 1st November 2011–31st October 2012). Members of *Enterobacteriaceae* and non fermenting Gram negative rods were obtained from Silchar Medical College and Hospital, Silchar, India. Screening and molecular characterization of β-lactamase genes was done. Integrase gene PCR was performed for detection and characterization of integrons and cassette PCR was performed for study of the variable regions of integron gene cassettes carrying *bla*_OXA-10_. Gene transferability, stability and replicon typing was also carried out. Isolates were typed by ERIC as well as REP PCR.

**Results:**

Twenty-four isolates of Gram-negative bacilli that were harboring *bla*_OXA-10_ family (OXA-14, and OXA16) with fact that resistance was to the extended cephalosporins. The resistance determinant was located within class I integron in five diverse genetic contexts and horizontally transferable in *Enterobacteriaceae*, was carried through IncY type plasmid. MIC values were above break point for all the tested cephalosporins. Furthermore, co-carriage of *bla*_CMY-2_ was also observed.

**Conclusion:**

Multiple genetic environment of *bla*_OXA-10_ in this geographical region must be investigated to prevent dissemination of these gene cassettes within bacterial population within hospital settings.

**Electronic supplementary material:**

The online version of this article (doi:10.1186/s13104-017-2467-2) contains supplementary material, which is available to authorized users.

## Background

Expansion of β-lactamases in Gram-negative rods has been documented as most severe threat to the management of infectious diseases [1–4]. The ever-increasing use of antibiotics with the evolution of intrinsic and acquired resistance has led to the development of resistance mechanism in Gram-negative rods contributing to the expansion of several multi-drug resistance epidemics in hospital environment [1, 3, 5]. OXA-10 type was known to have narrow spectrum β-lactamase activity; although variant of this enzyme family has expanded-spectrum activity [3, 6, 7]. It has been extensively associated with infection of Gram-negative bacteria in the last two decades restricting therapeutic options. These genes are often reported to be located within integron gene cassettes [3, 4]. However, these rare types of beta lactamases are often unreported in the light of current incidence of New Delhi metallo beta lactamase and CTX-M types. The current study reports the dissemination of highly stable *bla*_OXA-10_ family among diverse group of nosocomial isolates of Gram-negative bacilli within a tertiary referral hospital of the northern part of India.

## Methods

### Sample size

A total number of 590 consecutive, non duplicate, Gram-negative rods consisting members of *Enterobacteriaceae* family (*Escherichia coli*, *n* = 208; *Klebsiella* spp., *n* = 99; *Proteus* spp., *n* = 28) and non fermenting Gram-negative rods (*Pseudomonas aeruginosa*, *n* = 241; *Acinetobacter baumannii*, *n* = 14) were collected from different clinical specimens spanning a period of 1 year (November 2011–October 2012) from Silchar Medical College and Hospital, India (Table 1).

**Table 1:** Clinical and molecular evidence of isolates harboring *bla*_OXA-10_ β-lactamase family

Sample number	Male/female	Wards/OPD	Types of clinical specimen	Isolates	Other β-lactamase genes/genotypes	Genetic environment	Plasmid replicon type		Strain typing
							Wild types	Transformants	
1.	Male	Surgery	Pus	*P. aeruginosa*	OXA-14	Type 4	FIC, Y	–	REP type 1
2.	Female	Gynecology	Urine	*E. coli*	CMY-2	–	FrepB, Y	Y	ERIC type 1
3.	Male	Medicine	Stool	*P. aeruginosa*	OXA-16	Type 1	Y	–	REP type 2
4.	Male	Surgery	Urine	*E. coli*	OXA-14	Type 3	Y	Y	ERIC type 2
5.	Female	Medicine	Urine	*E. coli*	OXA-14, SHV, CTX-M,	Type 4	Y	Y	ERIC type 3
6.	Female	Medicine	Urine	*E. coli*	OXA-16, SHV, CTX-M, TEM, CMY-2	Type 2	Y	Y	ERIC type 4
7.	Male	Surgery	Pus	*P. aeruginosa*	OXA-14, OXA-2, SHV	Type 5	FIB, Y	–	REP type 3
8.	Female	Pediatrics	Urine	*P. aeruginosa*	OXA-16, SHV, CTX-M	Type 5	FIC, Y	–	REP type 4
9.	Female	Surgery	Pus	*E. coli*	OXA-16, OXA-14, CTX-M	Type 3	Y	Y	ERIC type 5
10.	Male	Medicine	Urine	*E. coli*	OXA-14, CTX-M	Type 1	Y	Y	ERIC type 6
11.	Female	Medicine	Urine	*P. aeruginosa*	OXA-16, SHV, CTX-M, TEM	Type 4	FIB, Y	–	REP type 5
12.	Female	Pediatrics	Urine	*P. aeruginosa*	OXA-16, CTX-M, TEM	Type 4	FrepB, I1, Y	–	REP type 6
13.	Female	Surgery	Pus	*E. coli*	OXA-16, SHV, CTX-M, CMY-2	Type 5	Y	Y	ERIC type 7
14.	Male	Pediatrics	Oral swab	*Proteus* spp.	OXA-14	Type 3	Y	Y	ERIC type 13
15.	Male	Surgery	Pus	*P. aeruginosa*	OXA-14, CTX-M	Type 1	FIC, Y	–	REP type 7
16.	Female	Surgery	Pus	*E. coli*	OXA-14, SHV	Type 5	Y	Y	ERIC type 8
17.	Male	Surgery	Pus	*P. aeruginosa*	OXA-16	Type 3	FIB, Y	–	REP type 8
18.	Male	Surgery	Pus	*Klebsiella* spp.	OXA-14, SHV, CTX-M, CMY-2	Type 4	Y	Y	ERIC type 10
19.	Female	Gynecology	Urine	*P. aeruginosa*	OXA-16	Type 1	Y, K	–	REP type 9
20.	Male	Pediatrics	Urine	*P. aeruginosa*	OXA-14, SHV, CTX-M	Type 3	FIC, Y	–	REP type 10
21.	Female	Gynecology	Urine	*E. coli*	OXA-14, SHV, CTX-M,	Type 4	Y	Y	ERIC type 9
22.	Female	Medicine	Urine	*Klebsiella* spp.	OXA-14, SHV	Type 3	Y	Y	ERIC type 11
23.	Male	Medicine	Urine	*P. aeruginosa*	OXA-14	Type 4	FIB, Y	–	REP type 11
24.	Male	Medicine	Urine	*Klebsiella* spp.	OXA-14, SHV, CTX-M, TEM	Type 1	Y	Y	ERIC type 12

*OPD* outpatient department

### Screening and molecular characterization of β-lactamases

Isolates resistant to at least one of the expanded-spectrum cephalosporins (cefotaxime, ceftazidime, or ceftriaxone) were selected for the study. For amplification and characterization of β-lactamase genes, multiplex PCR was performed (T100, BioRad-USA) with set of five primers namely: *bla*_CTX-M_ [8], *bla*_TEM_, *bla*_OXA-10_, *bla*_OXA-2_ [9] and *bla*_SHV_ [10] (Additional file 1: Table S1). Previously confirmed beta-lactamase genes were used as positive control which were obtained from Institute of Medical Sciences, Banaras Hindu University, Varanasi, India. PCR was performed by using Go-Taq Green Master Mix (Promega, Madison, USA) and products were visualized in 0.5% Agarose gel. PCR product was purified using Gene JET PCR product purification kit (Thermo Scientific, Lithuania) and sequencing was done using Sanger’s Method in Xcelris Lab Pvt Ltd in Ahmedabad, India.

PCR assay was also carried out for detection of AmpC genes in donor strains and transformants as described earlier [11]. Carbapenemase production in donor strains and transformants was tested by modified Hodge test, Imipenem-EDTA disc test [12] and boronic acid inhibition test [13] followed by PCR assay targeting *bla*_OXA-48_ [14] and *bla*_OXA-23, -24/40 and -58_ [15].

### Study of genetic context and southern blot hybridization

Presence of integron was detected by integrase gene PCR [16]. To study the variable regions of integron gene cassettes carrying *bla*_OXA-10_, two PCR assays were performed consequently: in one reaction 5′-CS and reverse primer of *bla*_OXA-10_ and in other reaction 3′-CS and forward primer of *bla*_OXA-10_ were used [9, 16] (Additional file 1: Table S2). Purified PCR products were cloned on pGEM-T vector (Promega, Madison, USA) and further sequenced. To validate our study, Southern hybridization was performed on agarose gel by in-gel hybridization with the *bla*_OXA-10_ family specific probe labeled with Dig High Prime Labeling Mix (Roche, Germany) detection Kit. Plasmid DNA was separated on agarose gel and transferred to nylon membrane (Hybond N, Amersham, UK) and hybridized.

### Transferability, PCR-based replicon typing and stability of *bla*_OXA-10_ family

Transformation was carried out using *E. coli* JM107 as recipient. Conjugation experiment was performed taking clinical isolates as donors and a streptomycin resistant *E. coli*-strain B (Genei, Bangalore) as recipient and transconjugants were selected on Luria–Bertani Agar plates containing cefotaxime (0.5 µg/ml) and streptomycin (800 µg/ml). Plasmid transfer was confirmed by colony PCR of transconjugants and transformants with the targeted primers [8, 9]. Plasmid stability of all *bla*_OXA-10_ producers as well as their transformants was analyzed by serial passages method for consecutive 115 days without antibiotic pressure [17]. Colony PCR assay was carried out in the isolates after each passage. Incompatibility typing was carried out by PCR-based replicon typing targeting 18 different replicon types [18] among all the wild types and their transformants carrying *bla*_OXA-10._

### Antimicrobial susceptibility and minimum inhibitory concentration determination

Antimicrobial susceptibility of *bla*_OXA-10_-harboring donor strains as well as transformants was determined by Kirby–Bauer disc diffusion method towards all non-β-lactam antibiotics (Hi-Media, Mumbai, India) [19]. MICs of donor as well as transformants and transconjugants were also done against beta lactam groups on Muller Hinton agar (Hi-Media, Mumbai, India) plates by agar dilution method and results were interpreted as per CLSI recommendation [19].

### Strain typing

Typing of all *bla*_OXA-10_ harboring isolates was done by Enterobacterial repetitive intergenic consensus (ERIC) and repetitive extragenic palindromic (REP) PCR [20].

## Results and discussion

**Fig. 1:**
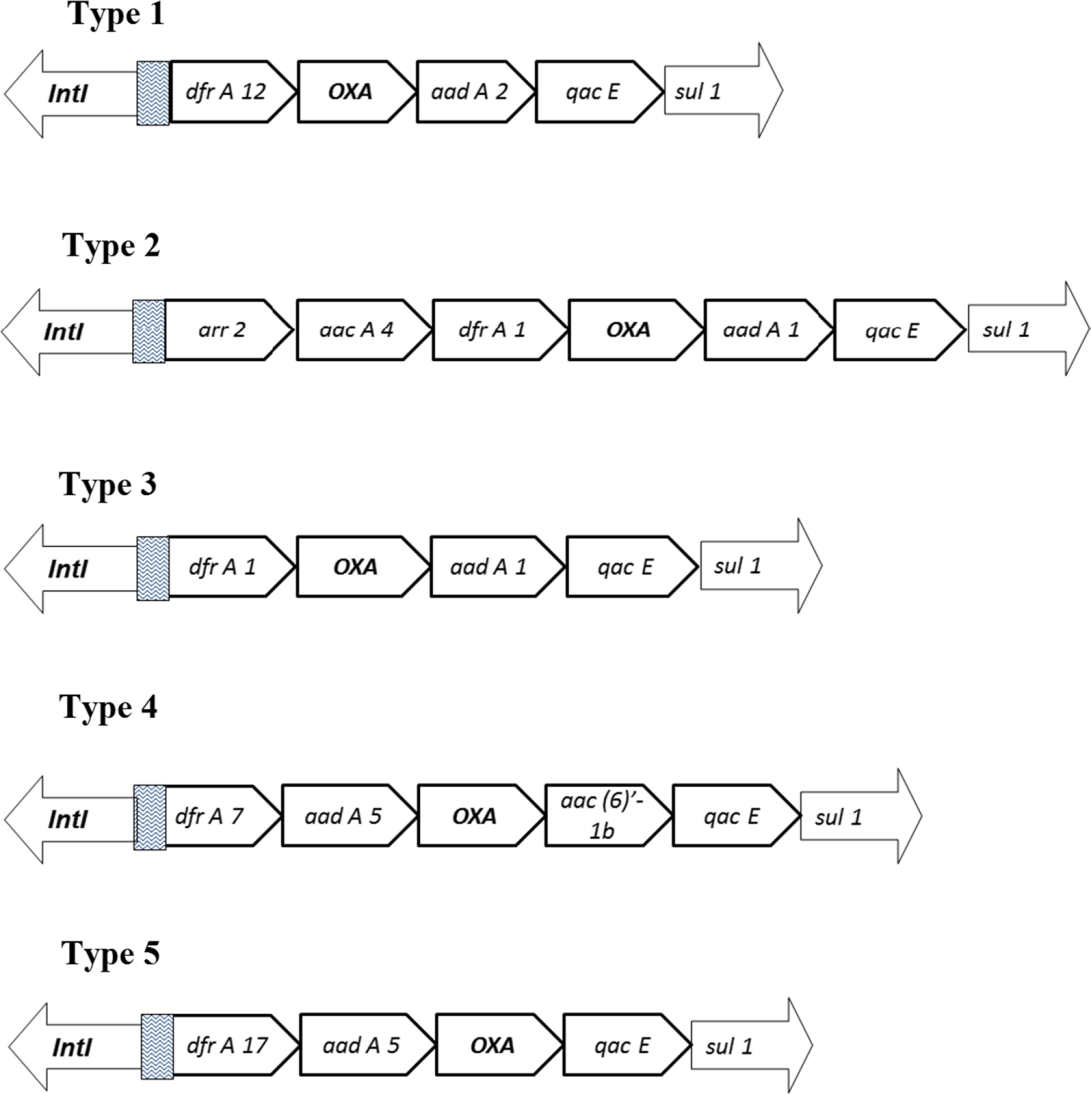
Schematic representation of variable region of class 1 integron types in OXA-10 producing Gram negative rods

A total of 58.5% (*n* = 345) isolates were resistant to expanded-spectrum cephalosporins. Among them 24 showed amplification with *bla*_OXA-10_ primers and were further confirmed by sequencing as OXA-14 (n = 15), and OXA-16 (n = 9) derivatives (Table 1). Sequencing results confirmed the presence of resistance determinant within class I integron with five different types of genetic environment (Fig. 1; Table 1). Upstream region of *bla*_OXA-10_ was occupied by *dfrA12* (Type 1)*, dfrA17* (Type 5)*, dfrA1* (Type 3 and Type 2)*, dfrA7* (Type 4)*, arr2* (Type 2), *aac A4* (Type 2)*, aad A5* (Type 4 and Type 5), while in downstream regions *aad A2* (Type 1), *aad A1* (Type 2 and Type 3), *aac (6′)1b* (Type 4), and *qacE* (Types 1–5) genes were present (Fig. 1). Plasmid encoding *bla*_OXA-10_ was successfully transferred in *E. coli* for *Enterobacteriaceae*, while in case of *P. aeruginosa* the attempt was not successful. Hybridization experiment revealed that *bla*_OXA-10_ carriage was plasmid mediated for *Enterobacteriaceae*. Replicon typing result established that *bla*_OXA-10_ was encoded within IncY type plasmid (Table 1) (Additional file 2: Figure S1). All isolates belonging Enterobacteriaceae family were found susceptible to tigecycline while Polymyxin B susceptibility was observed in *P. aeruginosa*. In case of *Enterobacteriaceae,* MICs of donor strain and transformants were observed above the breakpoint against cephalosporins, carbapenems, and monobactams (Table 2) and similar MIC pattern was too observed in *P. aeruginosa* (Table 3). The *bla*_OXA-10_ was highly stable and none of the isolates lost the gene till 115 serial passages. Modified Hodge test could detect carbapenemase activity in seven isolates and *bla*_CMY-2_ was also co-carried along with *bla*_OXA-10_ in four isolates (Table 1). DNA fingerprinting by ERIC (in *E. coli*—ERIC Types 1–9; *Klebsiella* spp.—ERIC Types 1–3; *Proteus* spp.—ERIC Type 1) (Table 1; Additional file 2: Figure S2) and REP PCR (In *P. aeruginosa*- REP Types 1–11) (Table 1; Additional file 2: Figure S3) was suggestive that diverse clonal types were present.

**Table 2:** MICs range of *bla*_OXA-10_ harboring isolates in members of *Enterobacteriaceae*, and their transformants

Sample number	Antibiotics (µg/ml)															
	IPM		MEM		ETP		ATM		CAZ		CTX		CRO		FEP	
	DS	TF	DS	TF	DS	TF	DS	TF	DS	TF	DS	TF	DS	TF	DS	TF
2	4	2	2	2	2	2	64	64	64	64	64	64	64	64	128	128
4	4	4	4	4	4	4	128	128	64	64	64	64	128	128	128	128
5	4	4	4	4	4	4	256	256	128	128	64	64	128	128	128	128
6	4	4	4	4	4	4	256	256	128	128	128	128	128	128	128	128
9	2	2	2	2	2	2	512	512	128	128	128	128	128	128	128	128
10	2	2	2	2	2	<2	512	512	128	128	128	128	256	256	256	256
13	4	4	4	4	4	4	256	256	128	128	128	128	128	128	256	256
14	2	2	2	2	2	2	512	512	256	256	256	256	256	256	256	256
16	2	2	2	2	2	<2	256	256	256	256	128	128	256	256	256	256
18	4	4	4	4	4	4	512	512	128	128	128	128	128	128	128	128
21	4	4	4	4	4	4	256	256	256	256	256	256	256	256	256	256
22	4	4	4	4	2	2	256	256	256	256	128	128	256	256	256	256
24	2	2	2	2	<2	<2	256	256	128	128	128	128	256	256	256	256

*DS* parent strain, *TF* transformants, *IPM* imipenem, *MEM* meropenem, *ETP* ertapenem, *ATM* aztreonam, *CAZ* ceftazidime, *CTX* cefotaxime, *CRO* ceftriaxone, *FEP* cefepime

**Table 3:** MICs range of *bla*_OXA-10_ harboring *P. aeruginosa*

Sample number	Antibiotics (µg/ml)							
	IPM	MEM	ETP	ATM	CAZ	CTX	CRO	FEP
1	4	2	2	512	128	128	128	128
3	4	4	4	256	256	128	128	256
7	4	4	2	256	256	256	256	256
8	4	2	<2	256	256	128	128	128
11	4	4	4	512	256	256	256	256
12	2	2	<2	256	256	256	256	256
15	<2	<2	<2	256	128	128	128	128
17	4	4	4	512	256	256	256	256
19	2	2	<2	256	128	128	128	128
20	4	4	2	128	64	64	64	64
23	4	4	4	256	128	64	128	256
*E. coli* JM 107 without plasmid	<0.125	<0.125	<0.125	0.125	0.125	<0.125	0.125	<0.125

As transformation was not successful in *P. aeruginosa* MIC value of wild types are mentioned above

*IPM* imipenem, *MEM* meropenem, *ETP* ertapenem, *ATM* aztreonam, *CAZ* ceftazidime, *CTX* cefotaxime, *CRO* ceftriaxone, *FEP* cefepime

So far, in India predominant types of beta-lactamases are CTX-M-15 and in recent years carbapenem therapy is compromised due to emergence of New Delhi Metallo beta-lactamase from this subcontinent. However, OXA type beta lactamases with extended spectrum activities are rarely reported [21, 22]. Our data showed that the majority of the isolates were recovered from surgery ward (37.5%; *n* = 9) followed by medicine (33.33%; *n* = 8), pediatrics (16.66%; *n* = 4), and gynecology (12.5%; *n* = 3). Carriage of *bla*_OXA-10_ within integron with diverse genetic environment comprising different coexisting-resistant determinant shows its multiple sources of acquisition and complicacy while determining the therapeutic options. It was also found that *bla*_OXA-10_ was horizontally transferable in *Enterobacteriaceae* family which was supported by transformation and conjugation. However, unsuccessful transfer of *bla*_OXA-10_ in *P. aeruginosa* could be due to their plasmid not being replicated within *E. coli* recipient or a chromosomal location of the gene. High MIC against carbapenems could be due to presence of some gene types which was not amplified by our target primers. Capability of the organisms to retain the resistant gene even after withdrawal of antibiotic pressure underscores their vertical transfer and persistence in the cell, which possibly can be the reason of expansion of this resistance determinant within hospital environment as well as in community. High MICs of the donor strain as well as their transformants could be due to the coexistence of another β-lactamase enzyme as observed in the current study.

## Conclusion

To the best of our knowledge this is the first report of gene cassette-mediated carriage of *bla*_OXA-10_ from India. Their acquisition and dissemination as well as adaptation against high antibiotic pressure in the hospital environment demands immediate measure to prevent transmission of these genetic vehicles, by the adoption of proper infection control measures and treatment policies.

## Additional files

**Additional file 1: Table S1.** Oligonucleotides used as primers for amplification of different ESBL genes. **Table S2.** Primers used for characterization of integron.

**Additional file 2: Figure S1.** PCR detection of IncY (765 bp) in transformants plasmid harbouring *bla*_OXA-10._ Lane 1: Negative control; Lane 2-8: 765 bp IncY. **Figure S2.** DNA finger printing of *Enterobacteriace* by ERIC PCR. Lane L: 10 Kb DNA hyper ladder I; Lane 1–9: ERIC pattern of *E. coli* Types 1–9; Lane 10–12: ERIC pattern of *Klebsiella* spp. Types 1–3. Lane 13: ERIC pattern of *Proteus* spp. ERIC Type-1. **Figure S3.** DNA finger printing of *P. aeruginosa* by REP PCR, *P. aeruginosa* Rep Types 1–11.

## Abbreviations

ERIC: enterobacterial repetitive intergenic consensus
REP: repetitive extragenic palindromic
PCR: polymerase chain reaction
*bla*: beta-lactamase
Inc: incompatibility
MIC: minimum inhibitory concentration
EDTA: ethylene diamine tetra acetic acid
CLSI: Clinical Laboratory Standard Institute
