# Supplementary material for: Expansion of highly stable blaOXA-10 β-lactamase family within diverse host range among nosocomial isolates of Gram-negative bacilli within a tertiary referral hospital of Northeast India
Source: BMC Res Notes. 2017 Apr 4;10:145. doi: 10.1186/s13104-017-2467-2 (PMC5379701; doi:10.1186/s13104-017-2467-2)
Supplement: Supplementary file 1 — Additional file 1: Table S1. Oligonucleotides used as primers for amplification of different ESBL genes. Table S2. Primers used for characterization of integron. [file 13104_2017_2467_MOESM1_ESM.doc]

**Table S1**: Oligonucleotides used as primers for amplification of different ESBL genes

| **Primer pairs** | **Target** | **Sequence(5’ -3’)** | **Amplified Product size (bp)** | **Reference** |
| --- | --- | --- | --- | --- |
| TEM – F  TEM – R | TEM | ATGAGTATTCAACATTTCCG  CTGACAGTTACCAATGCTTA | 867 | Bert et al., 2002, J. A C. 50:11 – 18 |
| SHV – F  SHV – R | SHV | AGGATTGACTGCCTTTTTG  ATTTGCTGATTTCGCTCG | 392 | Colom et al., 2003, FEMS Microbiol let, 223: 147 – 151 |
| CTX – M – F  CTX – M – R | CTX – M –1,-2,-9 group | CGCTTTGCGATGTGCAG  ACCGCGATATCGTTGGT | 550 | Lee et al., 2005, J. A. C. 56:122 – 127. |
| OXA – 10 – F  OXA – 10 – R | OXA – 10 group | TCAACAAATCGCCAGAGAAG  TCCCACACCAGAAAAACCAG | 276 | Bert et al., 2002, J. A C. 50:11 – 18 |
| OXA – 2 – F  OXA – 2 – R | OXA – 2 group | AAGAAACGCTACTCGCCTGC  CCACTCAACCCATCCTACCC | 478 | Bert et al., 2002, J. A C. 50:11 – 18 |

**Table S**2: Primers used for characterization of integron

| **Primer** | **Nucleotide sequence (5' to 3')** | **Product size (bp)** | **Reference** |
| --- | --- | --- | --- |
| Int1F | CAG TGG ACA TAA GCC TGT TC | 160 | Koeleman et al., 2001. JCM, 39: 8-13. |
| Int1R | CCC GAG GCA TAG ACT GTA |
| Int2F | TTG CGA GTA TCC ATA ACC TG | 288 |
| Int2R | TTA CCT GCA CTG GAT TAA GC |
| 5’ – CS | GGC ATC CAA GCA GCA AG | -- |
| 3’ – CS | AAG CAG ACT TGA CCT GA | -- |
