# Supplementary material for: Expansion of highly stable blaOXA-10 β-lactamase family within diverse host range among nosocomial isolates of Gram-negative bacilli within a tertiary referral hospital of Northeast India
Source: BMC Res Notes. 2017 Apr 4;10:145. doi: 10.1186/s13104-017-2467-2 (PMC5379701; doi:10.1186/s13104-017-2467-2)
Supplement: Supplementary file 2 — Additional file 2: Figure S1. PCR detection of IncY (765 bp) in transformants plasmid harbouring bla OXA-10. Lane 1: Negative control; Lane 2-8: 765 bp IncY. Figure S2. DNA finger printing of Enterobacteriace by ERIC PCR. Lane L: 10 Kb DNA hyper ladder I; Lane 1–9: ERIC pattern of E. coli Types 1–9; Lane 10–12: ERIC pattern of Klebsiella spp. Types 1–3. Lane 13: ERIC pattern of Proteus spp. ERIC Type-1. Figure S3. DNA finger printing of P. aeruginosa by REP PCR, P. aeruginosa Rep Types 1–11. [file 13104_2017_2467_MOESM2_ESM.doc]

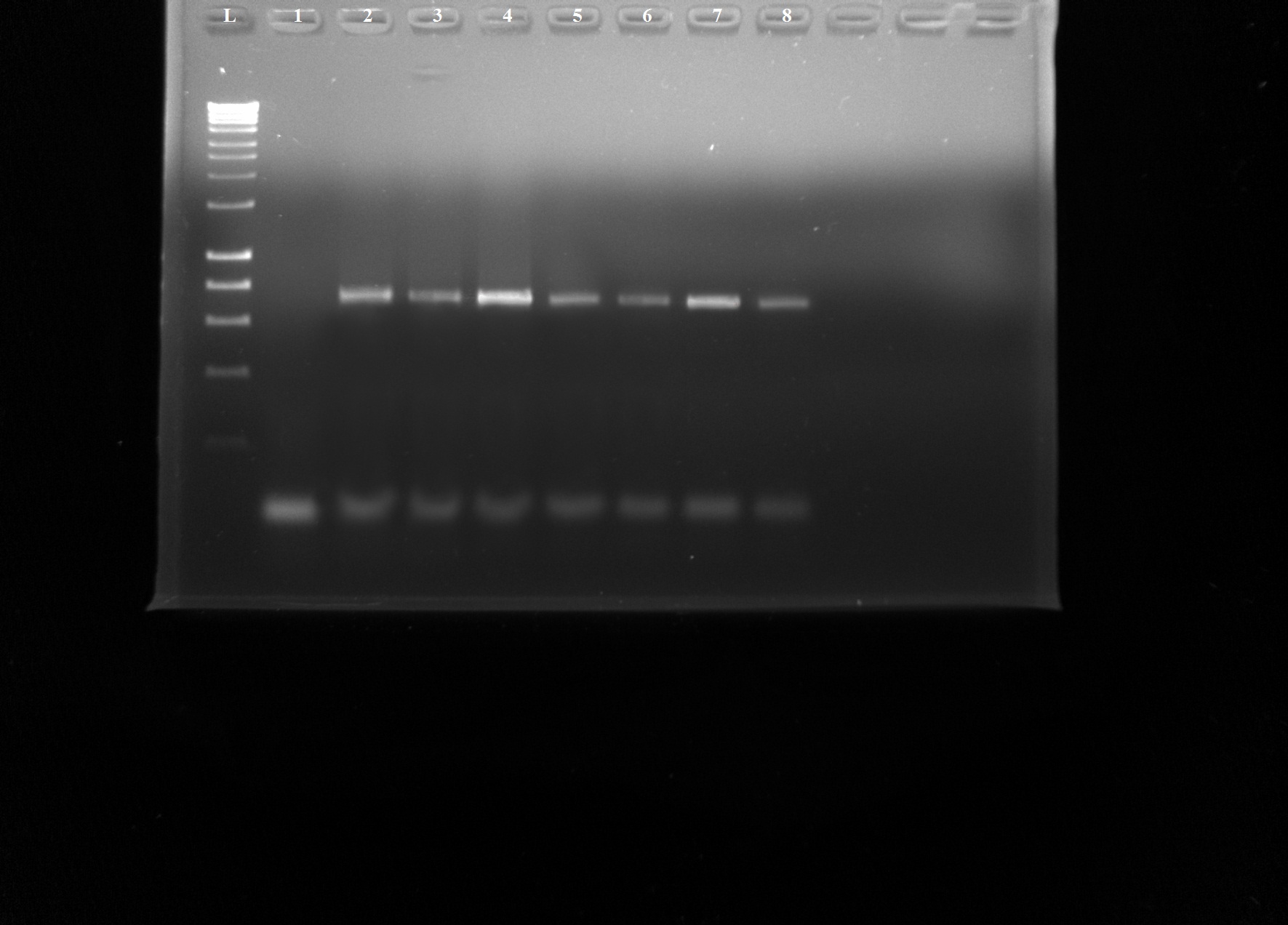


**Figure S1:** PCR detection of Inc Y (765 bp) in *E. coli* JM107 transformants plasmid harbouring *bla*_OXA-10_. **Lane 1:** Negative control; **Lane 2-8:** 765bp IncY ­­­­

**
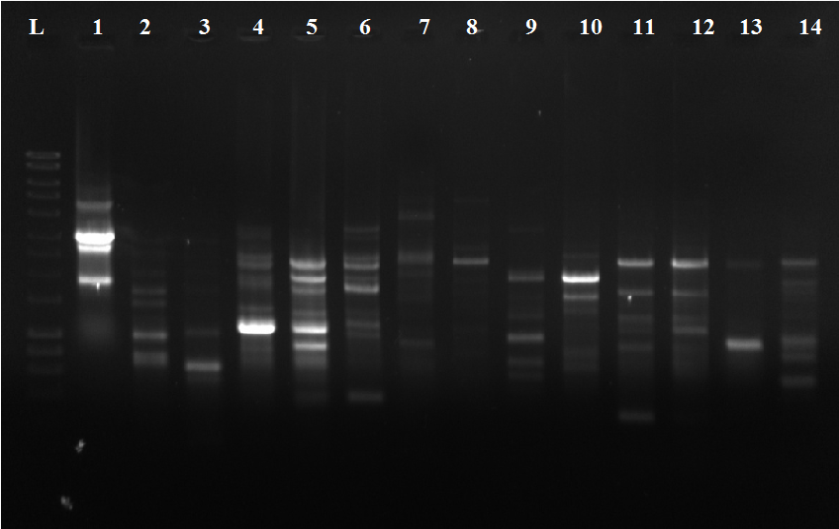
**

**Figure S2:** DNA finger printing of *Enterobacteriace* by ERIC PCR. **Lane L:** 10Kb DNA hyper ladder I; **Lane 1-9:** ERIC pattern of *E. coli* Type 1-9; **Lane 10-12:** ERIC pattern of *Klebsiella* spp. Type 1-3*.* **Lane 13:** ERIC pattern of *Proteus* Spp. ERIC type -1.

**
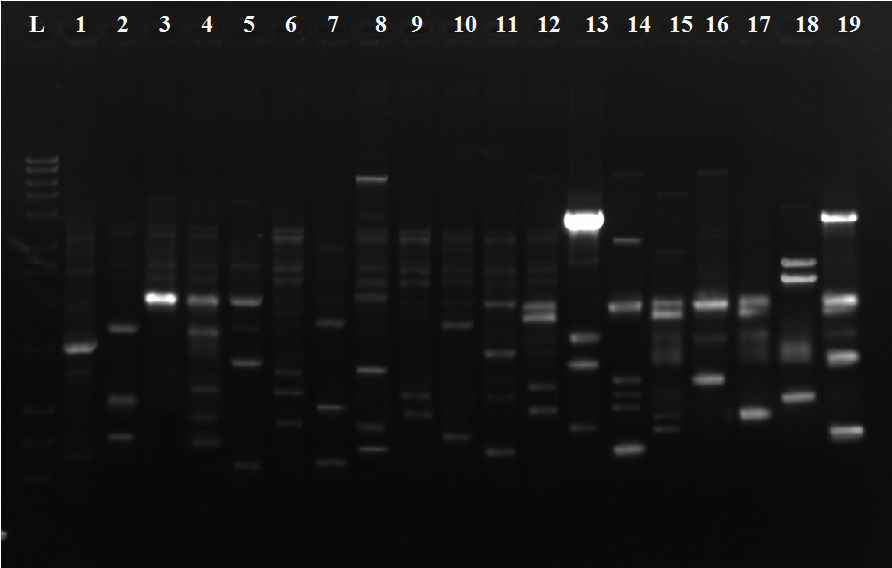
**

**Figure S3:** DNA finger printing of *P. aeruginosa* by REP PCR, *P. aeruginosa* Rep Type 1-11
